# Artificial intelligence in allergology: A Position Paper of the German Society of Allergology and Clinical Immunology (DGAKI), German Academy of Allergy, Clinical Immunology and Environmental Medicine (AeDA) and German Society of Pediatric Allergology and Environmental Medicine (GPA): Part 2 

**DOI:** 10.5414/ALX02655E

**Published:** 2026-11-05

**Authors:** Oliver Pfaar, Stephan Traidl, Friederike Bärhold, Sven Becker, Christoph Bergmann, Jeroen Buters, Stephanie Dramburg, Wojciech Francuzik, Michael Gerstlauer, Eckard Hamelmann, Camilo José Hernandez Toro, Ludger Klimek, Lars Lange, Paolo M. Matricardi, Laura Pfeffer, Mariel Suarez-Suarez, Philipp Utz, Martin Wagenmann, Margitta Worm, Mehrak Yoosefi, Sonja Mathes, Sebastian Seurig

**Affiliations:** 1 Department of Otorhinolaryngology, Head and Neck Surgery, Section of Rhinology and Allergy, University Hospital Marburg, Philipps-Universität Marburg, Marburg,; 2 Department of Dermatology and Allergy, Medizinische Hochschule Hannover, Hanover,; 3 Center for Rhinology and Allergology, Wiesbaden,; 4 Department of Otorhinolaryngology, Head and Neck Surgery, University of Tübingen,; 5 Department of Otorhinolaryngology, RKM 740 Interdisciplinary Specialist Clinic, Düsseldorf,; 6 Center of Allergy & Environment (ZAUM), Member of the German Center for Lung Research (DZL), Technical University and Helmholtz Center, Munich,; 7 Department of Pediatric Respiratory Care, Immunology and Intensive Care Medicine, Charité – Universitätsmedizin Berlin, Corporate Member of Freie Universität Berlin and Humboldt-Universität zu Berlin, Berlin,; 8 Allergology and Immunology, Department of Dermatology, Venereology and Allergology, Charité-Universitätsmedizin, Berlin,; 9 Pediatric Pulmonology and Allergology, University Hospital Augsburg, Augsburg,; 10 Children‘s Center Bethel, University Hospital OWL, University Bielefeld, Bielefeld,; 11 Institute of Biometry and Clinical Epidemiology, Charité – Universitätsmedizin Berlin, Berlin,; 12 Department of Pediatrics, GFO-Kliniken Bonn, St. Marien-Hospital, Bonn,; 13 Institute of Allergology, Charité – Universitätsmedizin Berlin, Corporate Member of Freie Universität Berlin and Humboldt-Universität zu Berlin,; 14 Fraunhofer Institute for Translational Medicine and Pharmacology ITMP, Allergology and Immunology, Berlin,; 15 Pediatric Dermatology and Allergology, Kinder- und Jugendkrankenhaus AUF DER BULT, Hannover,; 16 Kinderarztpraxis Hirschberg, Hirschberg,; 17 Department of Otorhinolaryngology, Düsseldorf University Hospital, Düsseldorf,; 18 Kinderarztpraxis Dr. med. Mehrak Yoosefi, Berlin,; 19 Department of Dermatology and Allergology, TUM Clinic Rechts der Isar, School of Medicine and Health, Technical University of Munich, Munich, and; 20 Department of Respiratory Medicine, Allergology and Sleep Medicine, General Hospital Nuremberg, Campus North, Paracelsus Medical University, Nuremberg, Germany; * Contributed equally as first authors.; ** Contributed equally as last authors.

**Keywords:** artificial intelligence, machine learning, allergology, immunology, CDSS, environmental analytics, patient education

## Abstract

Background: Although artificial intelligence (AI) is advancing in medicine, its use in allergology remains limited. Because allergic diseases are heterogeneous and clinical, environmental, and molecular data are difficult to integrate, assessment is needed. This second part of a position paper by German allergology societies and associations, led by the German Society of Allergy and Clinical Immunology (DGAKI), establishes a framework for AI in allergology. Materials and methods: The position paper is based on a narrative review and structured expert consensus and was developed in accordance with AGREE II principles. Part 2 comprises chapters 5 – 8, covering legal frameworks, ethical aspects, guiding principles for AI use, trends, and unmet needs. Results: AI use in allergology is regulated by the EU Medical Device Regulation and the EU AI Act. Most clinical applications are classified as high-risk systems requiring strict safety, transparency, and governance standards. Physicians remain responsible for deployment, with mandatory human oversight and integration of AI-related risks into quality management and patient communication. AI raises ethical challenges beyond technical and regulatory considerations. As no broadly accepted framework exists, core biomedical and AI ethics should guide evaluation. At the same time, AI may improve access through teleconsultations, support shared decision-making, and enable more patient-centered care by reducing documentation and triage workload. AI is expected to play a pivotal role in future allergology practice and research. Conclusion: Together with Part 1, this paper defines AI’s role, outlines methodological foundations, highlights initial applications, and provides a framework for evidence-based, patient-oriented integration requiring human oversight, scientific validation, and regulatory alignment.

## 5. National and international legal framework 

The use of AI in allergology, as is described in part 1 [45] and part 2 of this position paper, is embedded in a highly dynamic regulatory framework, primarily defined at the European level. For allergological practice, the guiding principle is that the new European Union AI Act (EU AI Act) [1] does not stand as an isolated law but must be read in conjunction with existing regulations, such as the Medical Device Regulation (MDR) [2]. This interlocking is intended to ensure that both the specific risks of algorithmic decision-making and the established safety standards for medical devices are fully covered. Even though Europe is leading the global stage with the AI Act, questions regarding practical interpretation remain unresolved [3]. 

### 5.1. The European Union AI Act 

Regulation (EU) 2024/1689 (EU AI Act) [1] has established the world’s first comprehensive legal framework for AI systems. The regulation follows a risk-based approach, categorizing AI systems according to their potential for harm. For allergology, it is of central importance that AI systems, which are either classified as a medical device themselves or serve as a safety component of one and require a conformity assessment by a Notified Body, are generally classified as high-risk AI systems according to Article 6(1) of the AI Act [1, 3]. This specifically includes software solutions for diagnostic support [4, 5] or for the creation of treatment plans [6], which are gaining importance in allergological diagnostics. 

In allergological practice, specialist physicians and medical institutions take on the role of the so-called deployer. This results in specific obligations under Article 26 of the regulation. A significant innovation, resulting from Article 4, obliges deployers to ensure sufficient AI literacy within the organization. This means that medical practice owners and clinical management must take measures to promote their staff’s understanding of the functionality, opportunities, and especially the risks of the systems used. 

Furthermore, the AI Act prescribes effective human oversight. Allergists must ensure that AI-supported recommendations can always be critically validated. If there is reason to believe that a system poses a risk to health, safety, or fundamental rights, the deployer is obliged to immediately suspend its use and inform the relevant market surveillance authorities and the provider. These duties are complemented by extensive transparency and information obligations towards patients when high-risk AI systems are significantly involved in clinical decisions. This should also be reflected in informed consent documents presented to patients. Sufficient AI literacy also gains importance in the context of patient education. 

A critical concern for clinical practice involves the legal implications of utilizing non-certified AI tools, such as general-purpose generative AI lacking a specific medical intended purpose, for diagnosis or treatment planning [6]. Under Article 16 of the MDR and the relevant provisions of the EU AI Act, any entity that modifies the intended purpose of an AI system or adapts a non-medical tool for a clinical function may be legally reclassified as the manufacturer (or “provider” under the AI Act) [1, 2]. This shift in legal status mandates that the clinician or medical institution assume full responsibility for regulatory compliance, including the performance of a formal conformity assessment and the maintenance of comprehensive technical documentation. Consequently, the practitioner carries the entire civil and professional liability for any diagnostic or therapeutic inaccuracies resulting from the use of such non-certified algorithms (Table 1). 

### 5.2. Medical Device Regulation and technical interaction 

Regardless of the new AI regulation, Regulation (EU) 2017/745 [2] (MDR) remains the primary framework for the safety and clinical performance of software in allergology. Software is considered a medical device if it pursues a medical intended purpose, such as risk assessment for the development of anaphylaxis or the analysis of sensitization patterns. Classification is based on the criteria of Rule 11 (Annex VIII) of the MDR, according to which software for diagnostic or therapeutic purposes is generally assigned at least to Class IIa [3]. 

To avoid administrative duplication, the legislator provides for extensive integration of regulatory processes. Manufacturers can integrate the AI Act’s requirements for quality and risk management into their existing MDR processes, which should result in a single set of technical documentation and a joint EU declaration of conformity. Also noteworthy is the demarcation from the Cyber Resilience Act [7]: medical devices are excluded from its scope, as the MDR already sets specific and extensive requirements for IT security and resilience. 

### 5.3. Monitoring structures and governance 

The monitoring of regulatory compliance follows a sector-specific approach. For AI systems that are simultaneously medical devices, the market surveillance authorities already established under the MDR remain responsible: in Germany, primarily the Federal Institute for Drugs and Medical Devices (BfArM) [3] and the respective state authorities. In the future, these authorities will monitor the requirements of both regulations in a unified capacity. Oversight of data protection, particularly regarding the processing of sensitive health data according to Article 9 GDPR [36], remains with the competent data protection authorities. 

The European Health Data Space (EHDS) [37] will introduce additional rules for the interoperability of electronic health record systems and wellness apps. Specific tasks within the framework of the EHDS, such as the enforcement of access rights to electronic health data, are the responsibility of the yet-to-be-designated Digital Health Authorities. In the course of this, non-statutory but state-funded infrastructural harmonization efforts also exist (e.g., HL7, FHIR). 

At the European level, a new governance architecture has been established. The AI Office within the EU Commission serves as the central executive body, supported by the European Artificial Intelligence Board, in which representatives of the member states coordinate a uniform application of the law. The Medical Device Coordination Group (MDCG) retains its central role in the interpretation of the MDR and works closely with the new AI bodies. In the future, the EHDS will provide further impulses for interoperability and secondary use of health data, with newly designated Digital Health Authorities taking over the enforcement of these rights. 

### Conclusion 9 

In summary, AI regulation in allergology is now defined by the combined framework of the MDR and the EU AI Act. Most diagnostic or therapeutic AI applications qualify as high-risk systems, requiring compliance with strict safety, transparency, and governance standards. Allergists act legally as deployers and must ensure adequate AI literacy and use aligned with the intended medical purpose. Human oversight is a legal requirement, as clinical responsibility and liability remain with the physician. Incorporating AI risks into quality management and informing patients about AI use are essential for legally secure practice. 

## 6. Ethical aspects of AI in allergology 

The increasing integration of AI into allergological care raises ethical questions that extend beyond technical performance and regulatory compliance. This chapter therefore outlines core principles of biomedical and AI ethics that are particularly relevant to allergology. These principles serve as a conceptual framework for evaluating AI-supported diagnostic, prognostic, and therapeutic applications. On this basis, allergology-specific ethical opportunities, risks, and future challenges are identified and discussed. 

Ethical reflection on the use of AI in clinical medicine is commonly grounded in the four core principles of biomedical ethics formulated by Tom L. Beauchamp and James F. Childress: respect for autonomy, beneficence, non-maleficence, and justice [8]. In the context of allergology, these principles provide a normative baseline for assessing AI-supported diagnostic, prognostic, and therapeutic applications. They allow ethical concerns to be systematically integrated into concrete clinical use cases [9]. 

A multitude of ethical deliberations have consequently led to a proliferation of principles for ethical AI, thereby augmenting these four principles with further concepts, including a widely known extension by explicability [10]. Importantly, explicability extends beyond technical model explainability to encompass institutional responsibility for governance and justification of AI use. Major organizations such as the World Health Organization (WHO) continue to publish widely recognized collections of ethical principles for AI in healthcare, introducing novel concepts like sustainability as an additional principle [11]. Such extensions reflect the recognition that classical bioethical principles alone may not sufficiently capture the sociotechnical characteristics of AI systems in healthcare. 

While these frameworks offer valuable guidance, their practical application in allergology raises specific ethical questions that are only partially addressed by general AI ethics. 

Allergology-specific considerations on AI ethics are scarce but are beginning to emerge [12]. As one of the first, Buckey et al. [9] provided a practice-oriented framework detailing steps for resolving ethical dilemmas in allergology and immunology; within allergological ethics, certain topics have received particular attention, particularly pediatric allergology and oral immunotherapy [13, 14, 15, 16]. Currently, no unified, broadly consented set of ethical principles for AI applications has been developed in allergology. In this situation, considering specific opportunities and risks of AI in allergology aids in detecting missing links in existing ethical frameworks. 

### Conclusion 10 

The increasing integration of AI into allergological care raises ethical challenges beyond technical and regulatory aspects. Primarily, key biomedical and AI ethical principles should be implemented as a framework for evaluating AI-supported diagnostic, prognostic, and therapeutic applications in allergology, highlighting specific opportunities, risks, and future challenges. As no broadly accepted ethical framework currently exists, analyzing these aspects in depth is fundamental to further development in this field. 

### 6.1. Chances of AI in allergology 

Against this ethical background, AI applications in allergology may offer several ethically relevant opportunities when deployed in concrete clinical contexts. 

From a fairness perspective, AI-supported teleconsultations and image-based decision support in conditions such as atopic dermatitis or acute urticaria can extend allergological expertise beyond specialized centers. This may reduce regional disparities in access to care, particularly for patients in rural or underserved settings. 

Patient autonomy may be strengthened through explicable AI systems that support shared decision-making in allergology [17]. AI-assisted interpretation of diagnostic results, such as sensitization profiles or symptom trajectories, can improve patient understanding of disease mechanisms and therapeutic options. When transparently integrated into clinical encounters, such systems may counteract paternalistic care models and facilitate informed, preference-based decisions. 

Beneficence in allergology is supported by AI applications that lower barriers to care and enable more individualized management strategies. By rapidly integrating guideline-based knowledge, real-world evidence, and patient-specific data, AI systems may assist in personalized treatment selection, for example in allergen immunotherapy (AIT) or biologic therapies. While in line with the maxime of salus aegroti suprema lex, the delegation of routine documentation tasks to AI may also relieve clinicians, lead to significant time savings, and allow greater focus on patient-centered interaction. 

Beyond shared decision-making, patient autonomy may also be enhanced through AI-assisted diagnostics. Machine learning approaches applied to longitudinal symptom data, imaging, or digital biomarkers may improve diagnostic sensitivity and specificity for allergic diseases while mitigating time constraints inherent to routine clinical practice. 

### 6.2. Risks and limitations of AI in allergology 

Despite their potential, AI applications in allergology hold ethical risks and limitations affecting recognized basic principles: autonomy, fairness, explicability, non-maleficence, and sustainability [18]. A central concern is the erosion of professional and patient autonomy through automation bias. In complex allergological decisions, such as differential diagnosis of multisystem allergic disease or treatment escalation, clinicians may over-rely on AI recommendations, even when these are based on incomplete or context-insensitive data [19]. Accordingly, localization of AI-driven clinical decision support systems (AI-CDSS) for allergy diagnosis has been advocated [20]. 

From a fairness perspective, algorithmic bias poses a further risk. As structural imbalances persist, distorted evidence bases lead to bias in algorithmic output. AI systems trained on non-representative datasets may underperform for marginalized groups, in patients with darker skin types or gender-specific aspects [21, 22]. Such biases risk reinforcing existing inequities rather than mitigating them. Closely related are concerns regarding data autonomy, profiling, and predictive risk scoring in allergy prevention or disease forecasting, particularly when patient consent and data governance remain intransparent [23]. 

Limited interpretability in algorithms represents an ethical constraint, conflicting with the principles of explicability and autonomy [12]. Many high-performing AI systems function as “black boxes” [24], with opacity potentially extending beyond successful implementation of black-box optimization techniques, like Bayesian optimization and evolutionary algorithms [24, 25]. Yet, AI-based CDSS need not be complete black boxes: in seasonal allergic rhinitis, high-performing CDSS for etiological diagnosis and AIT prescription have recently integrated useful techniques for interpretability and explainability using Shapley Additive Explanations analysis and other techniques [26]. Efforts toward explainable AI often entail a trade-off between interpretability and predictive performance, a tension that is particularly problematic in safety-critical allergological decisions where neither blind trust nor opaque accuracy is acceptable. 

In allergological research, the use of generative AI also raises concerns about data integrity, especially when using real-world patient data [27]. 

Non-maleficence is challenged by the known limitations of generative AI systems, including hallucinations and confident but incorrect outputs. In allergology, erroneous information regarding allergen avoidance, diagnostics, or therapy may directly – and potentially acutely – endanger patients, for instance with anaphylaxis. Regulatory frameworks therefore distinguish high-risk AI systems requiring stringent validation and oversight from lower-risk applications, while emphasizing continued human responsibility [1, 28]. 

However, human-in-the-loop approaches, although ethically necessary, may constrain scalability and limit anticipated gains in access to allergological care [29]. Moreover, human-in-the-loop applications presuppose adequate AI literacy among clinicians, which may further limit scalability in routine allergological care. 

Finally, sustainability constitutes a boundary condition. High energy consumption for computationally intensive AI queries, especially when simpler solutions suffice, raises environmental concerns that indirectly affect allergic disease burden. Ethical use of AI must therefore remain targeted, proportionate, and context-aware [30], also in allergology. 

### Conclusion 11 

AI applications in allergology may also hold ethical risks and limitations such as over-reliance on AI-guided recommendations, AI systems trained on non-representative or opaque datasets such as real-world patient data or limited introspection in data-driven algorithms. Generative AI may produce hallucinations and inaccurate outputs that can jeopardize patient safety in allergology. Consequently, high-risk AI systems require strict validation and oversight, with the ultimate responsibility remaining with clinicians. Lastly, environmental concerns regarding high ressource consumption exist. 

### 6.3. Upcoming ethical debates 

For research and publication, multiple stage-specific criteria have been formulated to ensure scientific rigor together with ethical soundness (TRIPOD-AI, STARD-AI, SPIRIT/CONSORT and SPIRIT/CONSORT-AI, DECIDE-AI) [31, 32, 33, 34], offering a structured assessment in study settings. With a growing body of literature on ethics and AI in allergology, allergology-specific ethical considerations become more visible in the scientific community and continue to be a paramount concern in daily routine and specialist allergology care. 

Looking ahead, ethical considerations will gain further importance as AI systems increasingly move from experimental settings into routine allergological care. The growing use of generative AI, real-world data, and adaptive learning systems will intensify questions of responsibility, transparency, and governance [35]. Continuous ethical reflection will therefore be essential to ensure patient safety, trust, and equitable access to innovation in allergology. 

### Conclusion 12 

As AI enters routine allergological practice, ethical challenges regarding accountability, transparency, and governance will intensify, requiring continuous ethical evaluation to ensure patient safety and trust. 

## 7. AI statutes in allergology: 7 requirements for artificial intelligence in allergological practice and research 

AI is poised to play a pivotal role in future allergology practice and research. With the following requirements, we draw together the conclusions of the preceding chapters, synthesizing current scientific evidence, regulatory requirements, and clinical experience. Through the requirements we propose a comprehensive and practice-oriented framework comprising seven core principles encouraging the diligent and effective use of AI in allergology. 

### Requirement 1: Taxonomy 

AI methods in allergology must be selected, evaluated, and applied in a context-aware and risk-sensitive manner. This requires explicit consideration of their underlying technology, along with its limitations, clearly distinguishing hypothesis-driven statistical models from prediction- and pattern-oriented machine learning approaches. It is essential to contextualize vision models and large language models (LLM) as task-specific, non-autonomous systems requiring domain expertise, validation across the full AI lifecycle, and effective human oversight. 

### Requirement 2: Augmented clinical decision-making 

AI methods for clinical decision support should be implemented in allergology exclusively as a physician-governed and supervised, human-in-the-loop tool to integrate complex and diverse allergological and environmental data in an evidence-based manner – thereby augmenting, not replacing human care. This aims to support individualized diagnostic and therapeutic decisions, while maintaining medical expertise, transparency, robust validation, and responsible integration into real-world clinical workflows. 

### Requirement 3: Patient-centered AI-adoption 

AI-based patient-facing tools, including LLM-driven chatbots and smart adherence technologies, should be deployed only under safety-by-design and transparency-by-design principles, with expert supervision, source-grounding, and comprehensibility. This aims to improve patient education, self-management, and adherence without exposing users to non-transparent, biased, or clinically hazardous misinformation. 

### Requirement 4: Alignment with a dynamic legal framework 

The legal framework for AI in allergology, defined by international regulations like the EU AI Act, the MDR, and national legislation, is established yet inherently dynamic; therefore, all healthcare stakeholders must maintain continuous regulatory awareness and AI literacy, recognizing their rights and obligations across the development, deployment, and use of AI systems that are increasingly integral to clinical care and biomedical research. 

### Requirement 5: Continuous ethical monitoring 

The development and use of AI in allergology must be ethically grounded in respect for patient autonomy, beneficence, non-maleficence, justice, and explicability. This requires trustworthy, fair, and supervised AI systems that improve care without undermining physician autonomy, patient trust, equity, or societal values, and that are continuously re-evaluated as technologies and clinical contexts evolve. 

### Requirement 6: Sustainability 

Sustainability constitutes an ethical and strategic opportunity in allergology when AI deployment remains targeted, proportionate, and indication-specific; the use of validated, purpose-built algorithms, rather than indiscriminate or general-purpose AI adoption, can limit unnecessary computational burden and align technological innovation with responsible resource use and long-term public health goals. 

### Requirement 7: Responding to trends and unmet needs 

To address persistent unmet needs in allergology, particularly limited access to expertise, insufficient personalization of diagnostics and therapy, and fragmented data landscapes, AI should be strategically developed as an enabling infrastructure for interoperable data ecosystems, scalable expertise, and precision-oriented care concepts (e.g., digital twins and task-specific decision support). This should be accompanied by sustained investment in AI literacy, interdisciplinary collaboration, and clinically anchored governance (Table 2). 

### Conclusion 13 

AI is poised to become a key enabler of future allergology practice and research. A comprehensive, practice-oriented framework integrates current scientific knowledge, regulatory frameworks, and clinical experience on AI as outlined in seven core requirements intended to support the responsible, effective, and patient-centered use of AI in allergology. 

## 8. Trends and unmet needs 

### 8.1. AI support for unmet needs in allergology 

Allergology is characterized by a high degree of clinical complexity, interindividual variability, and a growing gap between patient needs and available specialist care [38]. Unmet needs in allergological healthcare include limited access to expertise, insufficient availability of standardized test substances, fragmented interdisciplinary collaboration, and restricted therapeutic options, particularly with regard to efficacy, adherence, and long-term outcomes of AIT [39, 40]. AI-supported app-based interventions in patients with pollen allergy [41] demonstrate clinical benefits and highlight the potential of AI in allergological care, while AI has also contributed to advances in immunology research for decades [42]. 

Key unmet needs such as individualized diagnostics and allocation of AIT and symptomatic therapies, and scalable expertise may be partially mitigated through AI-driven approaches. In this context, AI should not be understood as a replacement for clinical judgment, but rather as a tool that enhances pattern recognition, integrates heterogeneous data sources, and supports evidence-informed decisions [40]. However, the effective and responsible deployment of AI in allergology requires a parallel development of AI literacy within the allergological community [27]. 

### 8.2. Similarities in trends for other areas of medicine 

Compared to other medical specialties, allergological care remains markedly under-personalized. While oncology has embraced AI-enabled stratification and personalized cancer therapies [43], allergology relies on comparatively uniform therapeutic approaches, exemplified by “one-size-fits-all” AIT preparations. 

Emerging AI-driven endotyping approaches aim to capture the immunological heterogeneity underlying allergic diseases and may serve as a foundation for more precise diagnostics and tailored interventions. Beyond clinical care, ML methods enable novel research strategies, including pattern discovery in high-dimensional immunological data, simulation of disease trajectories, and identification of new therapeutic targets [44]. 

### 8.3. Future patient journey 

Given the growing role of AI in allergology, future patient journeys are expected to be increasingly shaped by AI-supported systems, while remaining under continuous clinical supervision with a human-in-the-loop approach. We outline a vision of the future patient journey in which AI is integrated across different stages of care. Emerging clinical applications are likely to further expand the scope of allergological practice and give rise to new, AI-enabled patient pathways. In particular, opportunities arise from improved access to medical information and the continuous availability of AI-based support tools (Figure 1). 

A prominent trend in AI deployment is the development of chatbot-based systems serving both clinicians and patients. On the clinician side, AI-powered CDSS could assist with guideline-based recommendations, differential diagnosis, and therapy monitoring. While the potential benefits include efficiency gains and standardization, risks such as automation bias, lack of transparency, and medico-legal uncertainty must be carefully addressed. 

On the patient side, chatbots may support lay counseling, triage, and health literacy, provided that they adhere to professional standards and are clearly positioned as supportive, not substitutive, tools. Technically, such systems can be implemented via various architectures, including retrieval-augmented generation, which allows LLMs to generate responses grounded in validated medical knowledge bases. Similar approaches are already being applied in selected areas of precision medicine and could be adapted for allergology. 

### 8.4. Future potential and unmet needs 

Given the unmet needs in allergology patient care [38], allergology provides substantial opportunities for the implementation of AI-augmented approaches. Allergology and immunology do not yet fully leverage available AI applications and concepts successfully applied in other medical fields, such as digital twins [43]. The concept of the “digital twin” describes a virtual, data-driven representation of an individual patient that dynamically integrates clinical, biological, and environmental parameters [43]. In allergology, digital twins hold particular promise given the chronic, multifactorial, and context-dependent nature of allergic diseases. Potential applications include AI-supported diagnostics, such as phenotype and endotype classification, as well as therapy guidance and longitudinal monitoring. By integrating clinical history, laboratory findings, molecular sensitization profiles, and longitudinal outcome data, digital twins could support personalized risk assessment, therapy selection, and prediction of treatment response, including AIT. Moreover, they may support in silico modeling of test substances or immunological responses, partially compensating for limited availability of standardized allergens and facilitating hypothesis generation in translational research and complex networks. 

AI-driven networking, both technical and organizational, will be critical. To further unlock the potential of AI in allergology, isolated data sources must be transformed into interconnected ecosystems. This includes shared clinical-academic platforms, cooperative data usage agreements, and the systematic integration of registry data with routine clinical documentation. 

### Conclusion 14 

Unmet needs in allergological care include limited access to expertise, insufficient availability of standardized test substances, fragmented interdisciplinary collaboration, and restricted therapeutic options, particularly regarding AIT efficacy, adherence, and long-term outcomes. AI-driven approaches may help address key challenges such as individualized diagnostics, treatment selection, and scalable expertise. As AI assumes a growing role in allergology, future patient journeys are likely to be increasingly supported by AI systems while remaining under continuous clinical supervision through a human-in-the-loop approach. One promising concept is the digital twin, a virtual patient model integrating clinical, biological, and environmental data to support phenotype and endotype classification, therapy guidance, and longitudinal disease monitoring. 

## Authors’ contributions 

Oliver Pfaar: writing, conceptualization, development of design and methodology, review and editing. Stephan Traidl: writing, conceptualization, development of design and methodology, review and editing. Friederike Bärhold: writing, review, and editing. Sven Becker: writing, review, and editing. Christoph Bergmann: writing, conceptualization, review, and editing. Jeroen Buters: writing, visualization, review, and editing. Stephanie Dramburg: writing, conceptualization, review, and editing. Wojciech Francuzik: writing, conceptualization, review, and editing. Michael Gerstlauer: conceptualization, review, and editing. Eckard Hamelmann: writing, review, and editing. Camilo José Hernandez Toro: writing, conceptualization. Ludger Klimek: review and editing. Lars Lange: writing, visualization, review, and editing. Paolo M. Matricardi: conceptualization, review, and editing. Laura Pfeffer: writing, review, and editing. Mariel Suarez-Suarez: writing, visualization, review, and editing. Philipp Utz: writing, review, and editing. Martin Wagenmann: drafting, writing, conceptualization, review, and editing. Margitta Worm: writing, review, and editing. Mehrak Yoosefi: writing, conceptualization, review, and editing. Sebastian Seurig: writing, management and coordination (co-lead), conceptualization, development of design and methodology, review, and editing. Sonja Mathes: writing, management and coordination (co-lead), conceptualization, development of design and methodology, review, and editing. 

## Funding 

There was no external funding for this study. 

## Conflict of interest 

Oliver Pfaar reports grants and/or personal fees and/or travel support from AEDA, Alfried Krupp Krankenhaus, ALK-Abelló, Allergopharma, Almirall, Altamira Therapeutics, ASIT Biotech, AstraZeneca, Bencard Allergie GmbH/Allergy Therapeutics, Biologixpharma, Blueprint, Breazy Health, Cliantha, Deutsche AllergieLiga e.V., Cogitando GmbH/medcram, Deutsche Forschungsgemeinschaft, Dustri-Verlag, ECM Expro&Conference Management GmBH, Forum für Medizinische Fortbildung, Georg-Thieme-Verlag, GSK, HAL Allergy Holding B.V./HAL Allergie GmbH, Inmunotek, Ingress Health, Institut für Disease Management, IQVIA Commercial, Japanese Society of Allergology, Königlich Dänisches Generalkonsulat, Laboratorios LETI/LETI Pharma, Lilly, Lofarma, Medcram/Cogitando, Medizinische Hochschule Hannover, med update europe GmbH, Meinhardt Congress GmbH, Novartis, Paul-Ehrlich-Institut, Paul-Martini-Stiftung, PneumoLive, Pohl-Boskamp, Procter & Gamble, Red Maple Trials Inc., Regeneron, RG Aerztefortbildung, ROXALL Medizin, Sanofi Aventis, Sanofi Genzyme, Springer Publisher, Stallergenes Greer, streamedup! GmbH, Technical University Dresden, John Wiley & sons publishers, Wort & Bild Verlag, Verlag ME, Gemeinnütziger Verein zur Förderung der Allergiefortbildung (VFAF), all outside the submitted work. Oliver Pfaar is board member of the ENT section of the European Academy of Allergy and Clinical Immunology (EAACI) and member of the extended board of directors of the German Society of Allergy and Clinical Immunology (DGAKI); coordinator, main- or co-author of different position papers and guidelines in rhinology, allergology and allergen-immunotherapy; and he is Editor-in-Chief of Clinical Translational Allergy. 

Stephan Traidl has received honoraria for lectures or scientific advice from AbbVie, ALK, Almirall, Galderma, Janssen/JNJ, LEO Pharma, Lilly, Novartis, and Regeneron/Sanofi. 

Friederike Bärhold received speaker honoraria or advisory board fees from GSK, Bencard, AstraZeneca, Leti, and travel reimbursement from HAL Allergy. FB is involved in research sponsored by AstraZeneca, Sanofi, GSK, all outside the here-submitted work. 

Sven Becker has received lecture fees and participated in advisory boards of Allergopharma, Allergy-Therapeutics, ALK-Abelló, Ambu, AstraZeneca, Bencard Allergie, GSK, HAL Allergie, MSD, Sanofi, Smart Reporting, Stallergenes; and received research grants from Allergy-Therapeutics, BMBF, Akribion, Polyrizon, BRAIN AG, Pari, and Altamira AG. 

Christoph Bergmann received honoraria for presentations or advisory from Allergy Therapeutics, Bencard, Astra Zeneca, GSK, HAL Allergy, and SCS. 

Jeroen Buters has received honoraria for lectures from ALK, Allergopharma, Stallergenes, AEDA, and DGAKI. Other research funding was obtained from DFG, EU, and the Bavarian Government LGL. 

Stephanie Dramburg reports research funding from Thermo Fisher Scientific, speaker´s fees from Allergopharma, LETI, Bencard, dbv Technologies, OMRON Healthcare, Stallergenes, ALK, Siemens Healthineers, and is member of an advisory board for Bencard, as well as member of a data safety monitoring board for Allergy Therapeutics. 

Wojciech Francuzik declares no COI. 

Michael Gerstlauer is involved in studies with Allergopharma, Bencard Allergie, Boehringer, InfectoPharm, Stallergenes-Greer, has served at consulting for and received speaker honoraria from ALK-Abelló, Allergopharma, Bencard Allergie, Leti Pharma, Meda Pharma, Sanofi, Stallergenes-Greer, is part of specialist societies AGPAS, GPAU, GPP, DGKJ, EAACI, ERS, and is involved in AWMF guidelines for Allergen Immunotherapy, Insect Venom Allergy, Allergy Prevention, Severe Bronchial Asthma in Childhood, Allergic Rhinoconjunctivitis, S2k Bronchial Asthma, National Disease Management Guideline Bronchial Asthma, and the EAACI guideline on Allergen Immunotherapy. 

Eckard Hamelmann declares no COI. 

Camilo José Hernandez Toro declares no COI. 

Ludger Klimek has received research grants from Allergy Therapeutics/Bencard, United Kingdom/Germany; ALK-Abelló, Denmark; Allergopharma, Germany; ASIT Biotech, Belgium; AstraZeneca, Sweden; Bionorica, Germany; Biomay, Austria; Boehringer Ingelheim, Germany; Circassia, USA; Stallergenes, France; Cytos, Switzerland; Curalogic, Denmark; HAL, the Netherlands; Hartington, Spain; Lofarma, Italy; Viatris/Mylan, USA; Novartis, Switzerland; Leti, Spain; Roxall, Germany; GlaxoSmithKline (GSK), UK; and Sanofi, France, and/or has served as a speaker or worked as a consultant for the aforementioned pharmaceutical companies. LK is President of the Ärzteverband Deutscher Allergologen (AeDA), Vice President of the German Academy for Allergy and Environmental Medicine (DAAU), and a member of the Governance and NAIS Committees of the European Academy of Allergy and Clinical Immunology (EAACI); he is also editor-in-chief of *AllergoJournal* and *AllergoJournal International*. 

Lars Lange has received lecture fees and participated in advisory boards from Stallergenes, Allergopharma, Infectopharm, HAL Allergy, Thermo Fisher Scientific, DBV technologies, Novartis, Nestle Nutritional Institute, Nutricia, Sanofi, all outside the submitted work. 

Paolo M. Matricardi reports research support, speaker and/or consultancy fees from Allergopharma, Euroimmun, Hycor, MAD-X, Omron Healthcare, Stallergenes-Greer, Thermo Fisher Scientific, research support from TPS software production, speaker and/or consultancy fees from ALK-Abellò, Angelini, Bencard Allergie, HAL, all outside the submitted work. 

Laura Pfeffer declares no COI. 

Mariel Suarez-Suarez declares no COI. 

Philipp Utz has received honoraria for lectures or scientific advice from Aimmune, Allergopharma, ALK-Abelló, Berlin-Chemie, DAAB, Nutricia, PARI, and Sanofi-Regeneron. 

Martin Wagenmann has received lecture fees and participated in advisory boards of Allergopharma, ALK-Abelló, AstraZeneca, Celltrion, CSL Behring, Genzyme, GSK, HAL Allergie, MSD, NeilMed, Regeneron, Sanofi, Stallergenes; and received research grants from ALK-Abelló, AstraZeneca, EU, GSK, Novartis, Regeneron, Sanofi, Takeda. 

Margitta Worm reports honoraria and/or consulting fees from AbbVie, ALK-Abelló, Allergopharma, Almirall, Amgen, Boehringer Ingelheim, DBV Technologies, Genzyme, Kymab, LEO Pharma, Eli Lilly, Mylan (Viatris), Novartis, Pfizer, Regeneron Pharmaceuticals Inc., Sanofi, Stallergenes Greer. 

Mehrak Yoosefi declares no COI. 

Sebastian Seurig reports personal financial interests: ALK-Abelló (consulting, advisory board); AstraZeneca (lectures/training); Allergopharma (lectures); Bencard Allergy (advisory board). 

Sonja Mathes has received speaker honoraria from Thermo Fisher, ALK-Abelló, AstraZeneca and Bencard and travel reimbursement from ALK-Abelló and Bencard. 

## EQUATOR AGREE II 

Compliance with EQUATOR AGREE II statement. 

**Table 1.** Core obligations for specialist (allergological) deployers of high-risk artificial intelligence.

**Area**	**Requirement according to EU AI Act**
Personnel	Ensuring AI literacy (Art. 4)
Monitoring	Implementation of effective human oversight
Safety	Reporting of risks to market surveillance authorities
Patient rights	Information duty regarding the use of AI in decisions

**Table 2.** AI statutes in allergology: 7 requirements for artificial intelligence based on international, European, and national guidelines and regulations.

**Requirement**	**WHO guidance**	**EU guidance/regulation**	**German/national guidance**
Requirement 1: AI taxonomy	*WHO – Ethics and Governance of Artificial Intelligence for Health* (2021): context-dependent use, human oversight, risk awareness; *WHO – Responsible Use of AI in Health*	*Ethics Guidelines for Trustworthy AI* (High-Level Expert Group on AI, 2019): technical robustness, transparency, human agency and oversight; *EU AI Act* (Regulation (EU) 2024/1689) [1]: risk-based classification, non-autonomous high-risk AI systems	Deutscher Ethikrat: AI as supportive technology in medicine; Zentrale Ethikkommission bei der Bundesärztekammer (ZEKO): AI as decision support, not replacement of medical expertise
Requirement 2: Augmented clinical decision-making	*WHO – Ethics and Governance of Artificial Intelligence for Health*: human-in-the-loop, accountability; *WHO Global Digital Health Strategy 2020 – 2025*	*EU AI Act*: mandatory human oversight for high-risk AI (Art. 14); *Medical Device Regulation* (EU) 2017/745 [2]: clinical evaluation, intended medical purpose	ZEKO (Bundesärztekammer): final clinical responsibility remains with the physician; BfArM guidance on software as a medical device
Requirement 3: Responsible patient-facing AI	*WHO – Ethics and Governance of Artificial Intelligence for Health*: patient safety, autonomy, explainability, prevention of harm	EU AI Act: transparency and user information obligations; GDPR (Regulation (EU) 2016/679 [36]: patient rights, data protection; *Digital Services Act* (Regulation (EU) 2022/2065)	BfArM framework for digital health applications (DiGA); Deutscher Ethikrat: protection against misinformation and bias in digital health tools
Requirement 4: Alignment with a dynamic legal framework	*WHO – Responsible Use of Artificial Intelligence in Health*: governance, institutional capacity building	EU AI Act; Medical Device Regulation (EU) 2017/745 [2] GDPR; European Health Data Space (Regulation (EU) 2025/327^3^) [7]	National AI Strategy (Germany, updated); Digitalstrategie Deutschland; Gesundheitsdatennutzungsgesetz (GDNG)
Requirement 5: Continuous ethical monitoring	*WHO – Ethics and Governance of Artificial Intelligence for Health:* ethical governance, accountability, sustainability	*Ethics Guidelines for Trustworthy AI; EU AI Act:* risk management, post-market monitoring and corrective action	Deutscher Ethikrat: ongoing ethical evaluation of digital medicine; ZEKO: ethical responsibility in AI-supported clinical decision-making
Requirement 6: Sustainability	*WHO – Ethics and Governance of Artificial Intelligence for Health:* sustainability as an ethical principle	EU digital policy and emerging “Green AI” discourse; proportionality principle within EU technology governance	National AI Strategy (Germany): sustainable and responsible innovation; German research policy on resource-efficient digital technologies
Requirement 7: Responding to trends and unmet needs	*WHO Global Digital Health Strategy 2020 – 2025:* equity, access, system integration	*European Health Data Space:* interoperability and secondary use of health data; *Horizon Europe – Cluster Health*	Digitalstrategie Deutschland; registry- and routine-data integration under the Gesundheitsdatennutzungsgesetz (GDNG)

**Figure 1:**
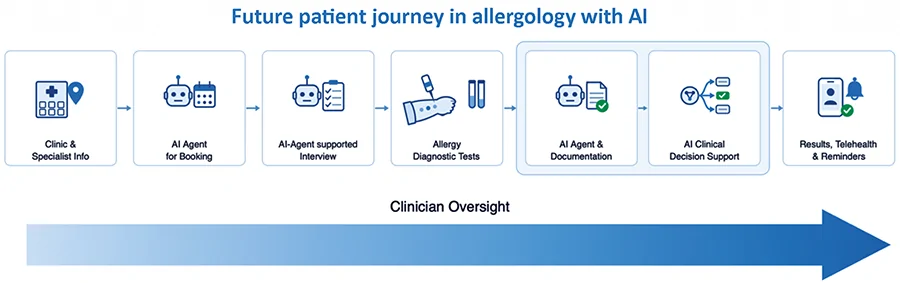
The patient journey could be accompanied by agentic AI systems that supplement, rather than replace, healthcare professionals across the continuum of care. Key stages include information search, AI-supported appointment scheduling, AI-enhanced history taking and medical documentation, clinical evaluation and diagnostic testing, AI-supported analysis and clinical decision support systems, discharge with structured follow-up, and continued follow-up facilitated by AI agents and appointment coordination. Throughout the journey, health experts work in close interaction with AI, actively monitoring medical data, interpreting results, and guiding clinical processes. Clinician oversight is required at all stages and becomes particularly critical when meaningful clinical data are generated, such as during history taking, laboratory diagnostics, and skin testing, and remains indispensable for all medical decision-making.

### Supplemental material

Supplemental material Compliance with EQUATOR AGREE II Checklist.
