# Supplementary material for: Artificial intelligence in allergology: A Position Paper of the German Society of Allergology and Clinical Immunology (DGAKI), German Academy of Allergy, Clinical Immunology and Environmental Medicine (AeDA) and German Society of Pediatric Allergology and Environmental Medicine (GPA): Part 2
Source: Allergol Select. 2026 Nov 5;10:323–36. doi: 10.5414/ALX02655E (PMC13750064; doi:10.5414/ALX02655E)
Supplement: Supplementary file 1 [file allergologieselect-10-323-S01.pdf]

Appendix (1)

Compliance with EQUATOR AGREE II Checklist

**AGREE Domain**	**Application**
Scope & purpose	Introduction
Stakeholder involvement	Author list / affiliations
Rigour of development	Methods + Delphi
Clarity of presentation	Structured chapters
Applicability	CDSS / Trends / Future
